# Pure exercise intolerance and ophthalmoplegia associated with the m.12,294G > A mutation in the *MT-TL2* gene: a case report

**DOI:** 10.1186/s12891-017-1781-0

**Published:** 2017-10-19

**Authors:** Patrick Soldath, Karen Lindhardt Madsen, Astrid Emilie Buch, Morten Duno, Flemming Wibrand, John Vissing

**Affiliations:** 1 Faculty of Health and Medical Sciences, University of Copenhagen, Copenhagen, Denmark; 2 Department of Neurology, Copenhagen Neuromuscular Center, Rigshospitalet, Copenhagen, Denmark; 3 Department of Clinical Genetics, Rigshospitalet, Copenhagen, Denmark

**Keywords:** Case report, Mitochondrial DNA, *MT-TL2* mutation, Mitochondrial myopathy, Exercise intolerance, Exercise physiological study, Maximal oxidative capacity

## Abstract

**Background:**

Pure exercise intolerance associated with exclusive affection of skeletal muscle is a very rare phenotype of patients with mitochondrial myopathy. Moreover, the exercise intolerance in these rare patients is yet not well explored, as most of known cases have not been assessed by objective testing, but only by interview. We report a patient with a mitochondrial DNA (mtDNA) mutation that gives rise to an exclusive myopathy associated with exercise intolerance and ophthalmoplegia. We quantified the patient’s exercise intolerance through detailed exercise testing.

**Case presentation:**

A 39-year-old man presented with exercise intolerance and chronic progressive external ophthalmoplegia. Sequencing of the entire mtDNA identified a m.12,294G > A mutation in the *MT-TL2* gene. The mutation was heteroplasmic in skeletal muscle (75%) while undetectable in blood, urinary sediment, and buccal mucosa as well as in tissues from the patient’s mother. The mutation affected a highly conserved site in the anticodon stem of the mitochondrial transfer RNA Leucine (CUN) molecule and lead to a severe combined respiratory chain defect. Exercise physiological studies in the patient demonstrated a significantly reduced maximal oxygen uptake of 20.4 ml O_2_ × min^−1^ × kg^−1^ (about half of normal) as well as threefold elevated lactate/pyruvate ratios.

**Conclusion:**

The findings of our study support that the m.12,294G > A mutation is pathogenic. Likely, the mutation arose sporadically in early embryogenesis after differentiation of the mesoderm into muscle progenitor cells, leading to a pure myopathic phenotype.

## Background

Point mutations in mitochondrial DNA (mtDNA) transfer RNA (MT-T) genes are associated with a wide variety of disorders that can affect virtually every tissue in the body. In the vast majority of cases, multiple tissues are affected simultaneously causing a multisystemic disease presentation, usually affecting highly oxidative tissues, such as the central nervous system and skeletal muscle, the most [1]. However, rarely a phenotype of isolated organ involvement is observed. In less than 1% of all cases of mtDNA point mutations, the phenotype is exclusively present in skeletal muscle, and presents as exercise intolerance and chronic progressive external ophthalmoplegia (CPEO) [2]. In most of these rare cases, the exercise intolerance has not been assessed by objective testing, but only by interview. Exercise intolerance can be quantified by measuring the maximal oxygen uptake (VO_2max_) as well as assessing resting and peak exercise-induced serum lactate levels and lactate/pyruvate ratios (L/P).

In this case report, we describe a patient with a previously reported m.12,294G > A mutation in the *MT-TL2* gene, who presents with pure exercise intolerance and CPEO. We performed detailed clinical, laboratory, and exercise physiological testing to describe the condition and present evidence that supports the pathogenicity of the mutation.

## Case presentation

### Patient

A 39-year-old man presented with a history of exercise intolerance that could be traced back to the first decade of his life. For example, during physical education classes in school he could not keep up with his classmates restricted by shortness of breath, muscle pain, fatigue, nausea, and even on some occasions vomiting from exhaustion. At age 39, he still complained of intolerance to physical exertion, and complained of persistent fatigue in his daily life, but was still able to perform all activities of daily living and work full-time as a maintenance engineer – a job of manually repairing industrial machinery. He had longstanding (> 15 years) bilateral ptosis that progressed and led to recent eyelid elevation surgery on his right eye. At the time of surgery, he was found to have external ophthalmoplegia and therefore referred to our mitochondrial clinic for further investigation for a possible mitochondrial disorder. He complained of no other symptoms than the fatigue and exercise intolerance. He had a past medical history of uncomplicated surgery for both umbilical hernia and bilateral tonsillitis in childhood. Family history was unremarkable. In particular, his mother, aged 64, was fully active with no signs of exercise intolerance or ptosis.

On examination, he had moderate, constant, and asymmetrical ptosis that was most evident on the left non-operated eye. There was prominent bilateral gaze palsy to 30° horizontally, 15° upwards, while downwards gaze was almost normal. He presented with generalized reduced muscle bulk, but without overt muscle wasting or weakness and with a height of 179 cm and weight of 73 kg. Neurological exam including motor, sensory, and coordination assessments, was otherwise normal. Resting serum creatine kinase (CK) was elevated at 1290 U/L (normal < 400) while acylcarnitine profile, and lactate, glucose, electrolytes, transaminases levels were normal. Furthermore, electrocardiogram and transthoracic echocardiogram were both normal.

### Muscle histology and enzyme histochemistry

**Fig. 1:**
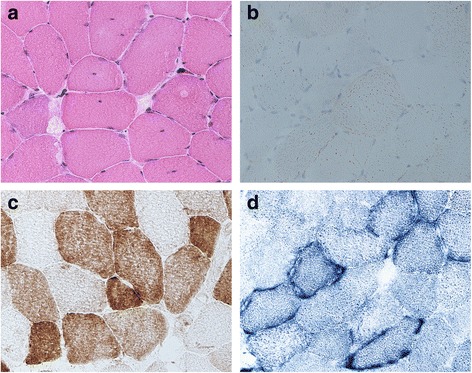
Muscle histology and histochemistry. **a** H&E stain revealing an enhanced variability in fiber size and some fibers showing internalized nuclei or increased subsarcolemmal eosinophilic accumulation. **b** Oil-Red-O stain revealing increased amount of lipid droplets. **c** COX histochemistry demonstrating a significant number of COX-deficient fibers. **d** SDH histochemistry showing multiple ragged-blue-fibers with subsarcolemmal accumulation of abnormal mitochondria

Based on his medical history and clinical presentation as well as laboratory findings we suspected a mitochondrial myopathy. After informed consent, a needle muscle biopsy of the vastus lateralis muscle was performed using a 5 mm Bergstrom needle. Standard histological and histochemical analyses were executed on fresh-frozen 10 μm sections according to established protocols [3]. Standard histology with hematoxylin and eosin (H&E) (Fig. 1a) and Oil Red O (Fig. 1b) stains revealed a myopathic picture involving enhanced variability in fiber size, multiple central nuclei, subsarcolemmal eosinophilic accumulation, and increased amount of lipid droplets. The fibers showing subsarcolemmal eosinophilic accumulation were found to be ragged-red fibers in Gomori trichrome stain (data not shown). Cytochrome c oxidase (COX) (Fig. 1c) and succinate dehydrogenase (SDH) (Fig. 1d) enzyme histochemistry revealed 32% COX-negative fibers and multiple ragged-blue fibers, respectively.

### Biochemical assay

Respiratory chain enzyme biochemistry was performed on muscle homogenate to assess the activities of the respiratory chain enzymes, following standard procedures [4]. This assay demonstrated a severe combined respiratory chain defect with significantly reduced activities of complexes I, III, and IV (residual activities 17%, 43%, and 32%, respectively) relative to citrate synthase (CS) when compared to 29 healthy age-matched controls (Table 1) [5]. In addition, CS showed a significantly increased activity of 205%.

**Table 1:** Activities of mitochondrial enzymes in muscle

	Patient	Controls mean ± SD [range] (*n* = 29)
Complex I/CS	0.06	0.34 ± 0.09 [0.18–0.58]
Complex II/CS	0.35	0.39 ± 0.07 [0.32–0.62]
Complex III/CS	0.51	1.20 ± 0.21 [0.75–1.71]
Complex IV/CS	1.2	3.8 ± 0.7 [2.5–5.7]
CS	634	309 ± 83 [181–468]

Enzyme activity is expressed as milliunits per milliunits citrate synthase (CS). CS activity is expressed as milliunits per milligrams protein. Reference values are from 29 age-matched controls and expressed as mean ± SD with ranges in brackets

### Molecular studies

**Fig. 2:**
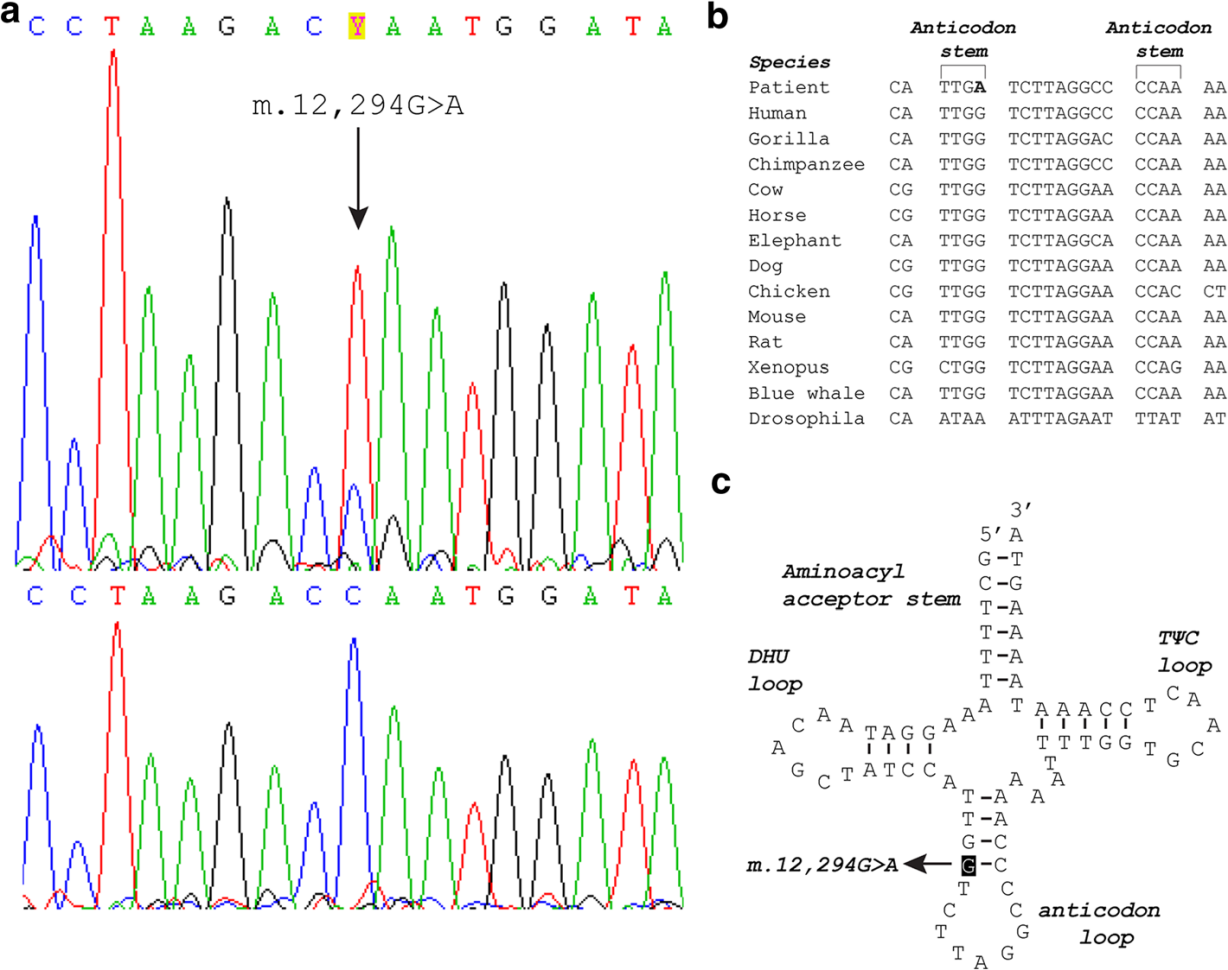
Molecular genetic investigation and functional consequence of the m.12,294G > A mutation. **a** Sanger sequencing electropherograms of *MT-TL2* from muscle (*top panel*) and buccal mucosa (*bottom panel*) encompassing the heteroplasmic m.12,294G > A mutation. The *arrow* in the *upper panel* marks the mutation. The mutation is not identified in the *bottom panel* and was likewise not detected in blood and urinary sediment. **b** Phylogenetic conservation of the anticodon stem and in between the anticodon loop of the mt-tRNA Leucine (CUN) sequence showing the high preservation of the affected base-pair throughout species. **c** Schematic representation of the mt-tRNA Leucine (CUN) cloverleaf structure showing the mutation in the anticodon stem and the affected G-C base-pair

To investigate the molecular basis of the combined respiratory chain defect, total genomic DNA including mtDNA was extracted from muscle as well as peripheral blood, buccal mucosa, and urinary sediment using standard methods [6]. The entire mtDNA was polymerase chain reaction (PCR) amplified in two fragments as previously described [7]. The fragments were then subjected to next generation sequencing with a mean coverage of > 1000 using updated chemistry and software described elsewhere [8]. Sequence analysis of mtDNA isolated from muscle identified a G-to-A transition at nucleotide position 12,294 in the *MT-TL2* gene that was present at a mutation load of 75% (Fig. 2a). The mutation was confirmed by direct Sanger sequencing (primers and conditions are available upon request). The mutation could not be detected in DNA isolated from urinary epithelial cells, buccal mucosa epithelial cells or leukocytes (NGS analysis to a mean coverage of >10,000). To investigate whether the mutation was maternally inherited or had arisen sporadically, the patient’s asymptomatic mother who requested testing was examined. After informed consent, the mother had a needle muscle biopsy performed as described for the patient, and investigated by PCR and direct Sanger sequencing of the muscle derived mtDNA. The mutation was not detected in the mother.

### Cycle ergometry

To measure the patient’s maximal oxygen uptake (VO_2max_), we had the patient perform an incremental cycle test to exhaustion as earlier described [9]. In order to assess the patients resting and peak exercise-induced serum lactate levels and lactate/pyruvate ratios (L/P), we drew 4 blood samples from the patient in relation to the cycle test and analyzed samples for serum concentrations of lactate and pyruvate. The first sample was drawn before the test with the patient sitting quietly at rest. The second sample was drawn when the patient became exhausted and could no longer pedal the bike. The third and fourth samples were drawn 5 and 10 min after the end of the test, respectively. The highest serum lactate concentration and highest fraction of concomitant serum lactate and pyruvate concentrations of the last three blood samples were considered the peak exercise-induced serum lactate and L/P, respectively. VO_2max_ was 20.4 ml O_2_ × min^−1^ × kg^−1^ and significantly reduced compared to sex- and age-matched healthy controls (~ 40 ml O_2_ × ^−1^ min × kg^−1^ ± ~ 12, mean ± 2SD) [10]. Resting serum lactate was 1.7 mmol × l^−1^, which was normal (0.7–2.1 mmol × l^−1^) and peak exercise-induced serum lactate was 9.4 mmol × l^−1^, which was similar to that found in 13 age-matched controls (10.4 mmol × l^−1^ ± 2.8, mean ± SD) [11]. Resting L/P was 30 and peak exercise-induced L/P was 71, which were highly elevated compared to 10 healthy individuals (< ~ 10 and < ~ 30, respectively) [12].

## Discussion

Pure exercise intolerance associated with exclusive affection of skeletal muscle is a very rare phenotype of patients with mitochondrial myopathy [2]. Such patients are often mistaken for having cardiopulmonary diseases due to the limitations in exercise performance. Here, we describe a patient who presented with exercise intolerance and CPEO associated with a m.12,294G > A mutation in the *MT-TL2* gene. In accordance with the exercise intolerance reported by the patient, VO_2max_ was reduced to about half of normal, indicating severely impaired aerobic capacity in skeletal muscle. In line with a perturbed aerobic metabolism, both resting and peak exercise-induced L/P were elevated threefold compared to normal. Resting serum CK was highly elevated, which is rare in mitochondrial myopathies [13], but a sign of significant limitation of mitochondrial function in skeletal muscle along with muscle morphology showing myopathic changes with damaged fibers, ongoing regeneration, mitochondrial proliferation, intra-muscular lipid accumulation, and abundant COX-negative fibers.

It is inherently difficult to distinguish pathogenic mtDNA mutations in MT-T genes from neutral polymorphisms, because they do not produce alterations in polypeptide sequences and at the same time the nature of mtDNA is highly polymorphic. In fact, recent studies have stressed that many reported mtDNA mutations affecting MT-T genes are likely to be neutral, polymorphic variants [14, 15]. Thus, it is important to assign definite pathogenicity to reported mutations by using the revised pathogenicity scoring system that evaluates the pathogenicity of mtDNA mutations affecting MT-T genes based on a list of canonical criteria [15]. In this case, there is substantial evidence that the m.12,294G > A mutation is pathogenic in regard to aforementioned scoring system and causes exercise intolerance and CPEO in the patient. Firstly, the biochemical assay of the respiratory chain enzymes showed significant impairment of complex I, III, and IV activities and CS activity was significantly increased as a measure of compensatory mitochondrial proliferation. Secondly, the mutation was heteroplasmic and most likely sporadic as it was present in post-mitotic tissue and undetectable in mitotic cells and in the mother. These two characteristics are in fact very common features of pathogenic mtDNA mutations [16, 17]. Thirdly, the mutation disrupts a Watson-Crick base pair that is phylogenetically highly conserved (Fig. 2b) in the anticodon stem of the mitochondrial transfer RNA (mt-tRNA) Leucine (CUN) molecule (Fig. 2c) and thus causes a conformational change of the molecule. Actually, a recent study found that this exact position in the anticodon stem of the mt-tRNA molecules is generally across all 22 mt-tRNA molecules associated with mutations that fulfill the canonical criteria for pathogenicity [18]. Fourthly, the mutation has been reported previously in a 45-year-old woman with the very same phenotype as our patient [19]. On histochemistry and biochemical assay she too had a large amount of COX-negative fibers and a severe combined deficiency of the respiratory chain complexes. Her mutation load was 59.8% in muscle, but 0% in blood and primary myoblasts. Additionally, single-fiber PCR analysis demonstrated that the mutation segregated with COX-negative fibers. Based on these findings the researchers concluded that the mutation most likely had arisen sporadically. Further support for pathogenicity of the mutation comes from the finding of a mutation affecting the neighboring nucleotide namely m.12,293G > A, which was reported to be sporadic with an occurrence exclusively in skeletal muscle at a mutation load of 41% giving rise to an axial myopathy in a 69-year-old woman [20]. Exercise capacity was not examined in these two other cases.

Considering our patient in relation to the two other cases it is striking that the mutations appear to be sporadic in origin, while at the same time only affect skeletal muscle. This suggests that these mutations are not maternally inherited, but arise sporadically in early embryogenesis after the differentiation of the mesoderm into muscle progenitor cells. This lack of transmission, and at the same time isolated organ involvement, is rare for mtDNA point mutations, but is often seen for mtDNA single, large-scale deletions that is a major cause of isolated CPEO and myopathy [21]. Sporadic mtDNA point mutations affecting only skeletal muscle have been previously observed in a few cases [22–24].

After our patient was diagnosed with his mitochondrial myopathy he was included in a randomized, double-blinded case-control study evaluating the safety of the drug omaveloxolone at various doses in patients with mitochondrial myopathies (clinicaltrials.gov). He was advised on following a fixed exercise regimen. He was not treated with any other drugs beside omaveloxolone or any supplements. At present, he is about to be included in another drug trial.

## Conclusion

Our study, together with the two previously reported patients affected in the same anticodon stem, adds to the evidence that the m.12,294G > A mutation is pathogenic in human mitochondrial disease. Likely, the mutation arose sporadically in early embryogenesis after differentiation of the mesoderm into muscle progenitor cells, leading to a pure myopathic phenotype.

## Abbreviations

CK: Creatine kinase
COX: Cytochrome c oxidase
CPEO: Chronic progressive external ophthalmoplegia
CS: Citrate synthase
H&E: Hematoxylin and eosin
L/P: Lactate/pyruvate ratio
mtDNA: Mitochondrial DNA
MT-T: Mitochondrial transfer RNA (gene)
mt-tRNA: Mitochondrial transfer RNA (molecule)
PCR: Polymerase chain reaction
SDH: Succinate dehydrogenase
VO_2max_: Maximal oxygen uptake
